# Ethnicity and Metabolic Syndrome: Implications for Assessment, Management and Prevention

**DOI:** 10.3390/nu12010015

**Published:** 2019-12-19

**Authors:** Scott A. Lear, Danijela Gasevic

**Affiliations:** 1 Faculty of Health Sciences, Simon Fraser University, Burnaby, BC V5A 1S6, Canada; 2 Division of Cardiology, Providence Health Care, Vancouver, BC V6Z 1Y6, Canada; 3 School of Public Health and Preventive Medicine, Monash University, Melbourne, VIC 3004, Australia; danijela.gasevic@monash.edu; 4 Usher Institute, University of Edinburgh, Edinburgh EH8 9AG, UK

**Keywords:** metabolic syndrome, ethnicity, prevention, lifestyle, cardiometabolic

## Abstract

The metabolic syndrome (MetS) is a constellation of cardiometabolic risk factors that identifies people at increased risk for type 2 diabetes and cardiovascular disease. While the global prevalence is 20%–25% of the adult population, the prevalence varies across different racial/ethnic populations. In this narrative review, evidence is reviewed regarding the assessment, management and prevention of MetS among people of different racial/ethnic groups. The most popular definition of MetS considers race/ethnicity for assessing waist circumference given differences in visceral adipose tissue and cardiometabolic risk. However, defining race/ethnicity may pose challenges in the clinical setting. Despite 80% of the world’s population being of non-European descent, the majority of research on management and prevention has focused on European-derived populations. In these studies, lifestyle management has proven an effective therapy for reversal of MetS, and randomised studies are underway in specific racial/ethnic groups. Given the large number of people at risk for MetS, prevention efforts need to focus at community and population levels. Community-based interventions have begun to show promise, and efforts to improve lifestyle behaviours through alterations in the built environment may be another avenue. However, careful consideration needs to be given to take into account the unique cultural context of the target race/ethnic group.

## 1. Introduction

The metabolic syndrome (MetS) is a constellation of cardiometabolic risk factors that results in an increased risk for type 2 diabetes (T2D), cardiovascular disease and premature mortality [1]. At its foundation is insulin resistance, in which the actions of insulin decrease, resulting in hyperinsulinemia. Left unchecked, insulin resistance can progress to MetS and prediabetes, and further to T2D. MetS is defined as the presence of at least three of the following five common clinical measures, which occur in people with insulin resistance: elevated triglycerides (TG), low high-density lipoprotein cholesterol (HDL-C), elevated blood sugar, elevated blood pressure (BP) and elevated waist circumference (WC) (Table 1, Table 2) [2].Within primary care and other front-line care environments, the MetS is a simple tool with which to identify people with insulin resistance and prediabetes, and therefore, at early risk for T2D and cardiovascular disease. As a result, it provides an opportune time for primary care providers to intervene and prevent progression to overt disease.

Despite the ease of assessment, the worldwide prevalence of MetS is not known [3,4], in part due to MetS not being a common clinical indicator, such as T2D, and thus not widely assessed. Further complicating estimates is the variation in definitions used in countries before, and even since [5], the MetS definition was harmonised in 2009 [2]. However, estimates suggest the worldwide prevalence of MetS to be 20%–25% of the population, based on a presumed prevalence threefold higher than T2D [4]. As the prevalence of obesity and T2D is expected to rise, so too is the prevalence of MetS. In countries where prevalence data do exist, it is clear that it differs by country. For example, the estimated prevalence of MetS in the United States is 33.4% [6], while in China, it is 14.4% [7].

The difference in MetS prevalence across countries is likely the result of different governmental, institutional and sociocultural factors at the population level, which can affect a range of upstream determinants including, but not limited to, the type of available foods and access, health care policies, education, employment and the physical environment. This is in addition to individual factors such as biology/genetics and sociocultural aspects, which are also likely relevant to the different prevalence of MetS between countries. These latter aspects may be collectively referred to as ethnic or racial differences, as many of these characteristics often cluster in specific and identifiable populations. Even within the same country, in the same local environment, the prevalence of MetS differs along certain predefined racial/ethnic groups. For example, in the United States, the prevalence of MetS is highest in Hispanics and lowest in African-Americans, with the prevalence of MetS in whites in between the two [8]. (It is important to recognise that the term “white” does not describe an ethnicity or ethnic group. The use of the term “white” in this article is only used when the authors of the original article that has been cited have used this term to describe one of their study populations without providing additional information on that group’s ethnic origins.) While recent national level data in Canada have not been reported, an earlier study reported MetS to be highest in people of Indigenous ancestry, followed by South Asians, Europeans and East Asians [9]. In Singapore, MetS is highest in South Asians, followed by Malays and then people of Chinese background [10]. Understanding the influence race/ethnicity has with respect to MetS is important for its proper assessment, treatment and prevention.

Ethnicity and race are fluid constructs that have no clear-cut definition, which are often (and incorrectly) used interchangeably. Despite these challenges, ethnicity and race are still used in medical literature as a means to differentiate between populations and recognise such differences in health management and disease prevention. Furthernore, ethnic and racial groupings often differ across the medical literature based on the research purpose, methods of data collection and even the lens of the researchers themselves. While individuals may ascribe their ethnicity within sociocultural aspects, medical guidelines such as those for the MetS attempt to define ethnicity primarily on common biomedical (whether biological or genetic) and/or geographical aspects. These are generally broad classifications, which often group multiple ethnic and racial groups into one. Sometimes this is based exclusively on geographical origins (for example, “Asian” or “European”) without recognising the ethnic or racial heterogeneity within these groupings and thus incorrectly inferring that the populations are homogeneous. While some biomedical characteristics align with certain ethnic and racial groups, this should not be interpreted that ethnicity and race are biomedical constructs, nor should ethnic and racial classifications be interpreted to reflect genetic variation.

It should also be noted that ethnicity and race are not the same. Race infers some biological foundations for differences among groups, while ethnicity views populations from a more social/cultural lens. However, these terms may be used in medical literature as if they are the same. Across the many papers cited in this review, some authors grouped study participants into categories they termed “race”, while others used groupings termed “ethnicity”, commonly without defining the terms or the categories. This lack of consistency makes it challenging to compare and contrast across the various studies. As a result, for the sake of this review, the terms are combined (albeit not ideally) into one called “race/ethnicity”, and when citing research, we have used the same race/ethnicity group names as the original authors.

## 2. Assessment

Of the five cardiometabolic risk factors, four of the definition thresholds apply to all race/ethnic groups. The fifth, WC, entails different threshold values based on race/ethnic background (Table 2). These differing thresholds are based on evidence that the association between WC, an indicator of abdominal obesity, and risk of cardiometabolic diseases differs by race/ethnicity. For example, at a similar WC, people of Chinese and South Asian background have higher values of total cholesterol and other cardiometabolic risk factors compared with people of European background [11,12,13]. If the goal is to identify people at the same level of cardiometabolic risk, lower WC thresholds for people of Asian background were created.

The requirement for lower WC thresholds in some racial/ethnic groups has its foundation in the differences in visceral adipose tissue (VAT). In particular, people of Asian backgrounds (South Asian, East Asian, Southeast Asian) have higher amounts of VAT at a given body size and WC [14]. For South Asians, this higher amount of VAT accounts for much of their elevated cardiometabolic risk compared with European-derived populations [15]. In African-Americans, VAT tends to be lower than whites at a similar body size [16]. Despite a higher prevalence of obesity and T2D [17,18], levels of VAT in North American Indigenous populations appear to be similar to that of Europeans of the same size [14,19].

While the use of race/ethnic-specific WC thresholds is reflective of the differing risk in different racial/ethnic groups, it can pose a challenge during assessment of MetS. This is reflected in the above biological foundations for having different WC thresholds for MetS. It must be acknowledged that the MetS uses a broad definition of race/ethnicity and population grouping based on geographical location, which does not reflect the many varying races and ethnicities within those groupings. There is even limited agreement in how race/ethnicity is defined [20]; however, it is probably best defined by self-report, meaning, each individual is likely to provide the most “accurate” identification of their own race/ethnicity.

In predominantly homogeneous populations found in many parts of Asia, the use of race/ethnic-specific WC thresholds should not pose a challenge [21]. However, in diverse populations such as in Europe and the Americas, determining the race/ethnicity of a patient can be challenging [22]. While self-identification may be the best indicator of race/ethnicity, this may be a conversation in which health care professionals may feel uncomfortable engaging. In addition, people who descend from a different geographical or race/ethnic region from where they currently live may identify more with the local context than their ancestral one. This may result in a conflict between how an individual identifies his/herself and how race/ethnicity is defined in the health system. In many locations, people may also identify with more than one race/ethnicity. Guessing on the part of the health care professional may be no better. The increasingly common prevalence of offspring from mixed ethnic partnerships can further complicate the matter [23], as there are no studies on how to apply the race/ethnic-specific WC thresholds to these individuals.

## 3. Treatment

As MetS allows for the presence of overt risk factors such as hypercholesterolemia, hypertension and T2D, when present, these should be treated as per local clinical guidelines. For patients with MetS but without overt risk factors, treatment through lifestyle therapy is effective at reversing MetS. In the Diabetes Prevention Program (55% white, 20% African-American, 16% Hispanic, 5% Native American and 4% Asian in the original trial [24], no analysis by race/ethnicity), the combined intervention of physical activity and diet resulted in a more than twofold reversal of the MetS compared with the placebo group and a 65% greater reversal rate than the metformin group [25]. A subsequent meta-analysis of combined physical activity and diet interventions found a twofold greater rate of MetS reversal compared with control interventions [26].

In isolation, regular aerobic physical activity of low-to-moderate intensity has resulted in reversal of the MetS in postmenopausal women (65% white, 30% African-American and 6% other in original trial [27], no analysis by race/ethnicity) [28] and reduction in MetS severity in a workplace setting using a wrist-worn activity monitor and smartphone app designed to enhance physical activity (conducted in Germany, race/ethnicity not reported) [29]. In addition to aerobic exercise interventions, a 12-week resistance training program was effective in reversing MetS in older women (race/ethnicity not reported) [30]. However, aerobic physical activity alone in men and women (56% white, 44% African-American) or a combination of aerobic and resistance physical activity may be superior to resistance training alone [31]. A randomised trial in Norwegian men and women focused on differing intensities of exercise on reversing the MetS is currently underway [32].

A secondary analysis in the PREDIMED randomised trial of participants with the MetS (approximately 97% European) at the study’s onset reported the Mediterranean diet resulted in an approximately 28%–35% reversal of MetS compared with the control diet [33]. However, due to later concerns with the PREDIMED methods [34] and republication with a smaller sample attesting to similar conclusions [35], the exact effect of the intervention may not be accurately reflected in this analysis.

Despite more than 80% of the world’s population being of non-European descent, the overwhelming majority of research on MetS is limited by the predominant focus on European-derived populations and a lack of race/ethnic-specific analyses. In terms of the benefits of interventions aimed at reducing or reversing MetS in other race/ethnic groups, few studies exist. Short-term diet studies have reported reversal of MetS in Iranians [36], South Asians [37] and people of Asian descent living in the United States [38]. A combined lifestyle intervention in Arabs living in Saudi Arabia reported a greater reduction in MetS compared with the group receiving general advice [39]. One randomised trial currently underway is investigating a combined lifestyle intervention of physical activity, nutrition improvement and weight loss in a diverse population in the United Kingdom [40], while another exercise-focused trial in African-American women with MetS is also in progress [41].

### 3.1. Comprehensive Lifestyle Interventions

Much information on reversing MetS may also be gleaned from randomised intervention studies that target individual components of the MetS or prevention/treatment of T2D. A small randomised study of Japanese men with MetS reported that a three-month intervention of diet and physical activity reduced WC and glycated haemoglobin, along with a nonsignificant reduction in MetS prevalence compared to control [42]. In African-Americans with T2D, a lifestyle weight loss intervention of 12 weeks resulted in lower weight, improved BP and glycaemic control compared with usual care after six months [43]. Similarly, a six-month weight loss program in African-Americans in the Southern United States resulted in reduction of WC and BP [44]. The Da Qing Diabetes Prevention Study in China reported reduced incidence of T2D by 45% following six years of a diet and physical activity intervention in people with glucose intolerance [45]. In Hispanic obese women, a community implementation of the Diabetes Prevention Program resulted in a greater decrease in WC and improved glycaemic control compared with metformin or standard care after 12 months [46]. In Brazil, a randomised intervention of lifestyle counselling resulted in improvements in WC and BP [47]. A nonrandomised study of translation of the Diabetes Prevention Program in Aboriginal people in the United States showed promise in reducing risk for T2D [48]. In Jewish and Bedouin women with post-gestational diabetes, a lifestyle counselling intervention resulted in reduction in glucose [49]. In South Asians living in the United Kingdom, compared with control, a diet and physical activity intervention resulted in improved WC but not glucose and BP after two years [50].

### 3.2. Nutritional Interventions

A wide variety of nutritional interventions ranging from macronutrient comparisons to supplementation with single foods or supplements have been carried out in a number of racial/ethnic groups. The most common dietary interventions are those focused on energy restriction in order to target weight loss, which generally improves cardiometabolic risk factors [51,52,53]. More recent attention has focused on the macronutrient combinations, and in particular, low-carbohydrate diets. A number of small randomised studies have indicated low-carbohydrate diets to result in more favourable improvements to glucose, insulin sensitivity, triglycerides and HDL-C [54], which may be independent of weight loss, suggesting a specific mechanism by which carbohydrates may promote cardiometabolic risk [55]. However, not all studies are in agreement and a meta-analysis of 23 randomised trials reported no difference in cardiometabolic risk factors between low-carbohydrate and low-fat diets [56].

In non-European-derived populations, diets with an emphasis on fruits and vegetables, healthy proteins and sodium reduction (such as the DASH diet) have demonstrated reductions in BP among African-Americans [57,58], East Asians [59,60] and South Asians [61,62]. For people with T2D, dietary interventions consisting of nutrition counselling following local guidelines for T2D care have resulted in improved glycaemic control, lipids and anthropometric measures in Arabs [63] and African-Americans [64]. Overweight and obese Malaysian adults undergoing a six-month trial of a high-protein, high-fibre diet had improvements to WC and glucose metabolism compared with control [65]. Studies in South Asians focusing on healthy protein, whether through meal replacement or nut supplementation, have reported reductions in glucose and WC, along with increases in HDL-C [61,62,66]. In Chinese men and women, a number of different randomised dietary interventions (high-protein, low-carbohydrate diets) have resulted in reductions in WC and lipid measures [67,68]. Diet supplemented with whole grain oats over six months reduced WC in Chinese men and women compared with control [69]. Very few studies have investigated interventions in Indigenous populations. A randomised study of flaxseed supplementation in Native Americans resulted in lowering low-density lipoprotein cholesterol (LDL-C) but did not affect HDL-C or TG [70], while a high-protein diet in Maori in New Zealand resulted in a greater decrease in WC compared with a low-fat or control diet [71].

### 3.3. Physical Activity Interventions

Numerous studies of various forms of physical activity have been conducted in a range of populations. In randomised trials, exercise interventions have demonstrated improvements in one or more of the components of MetS in South Asians [72,73,74] and East Asians [75,76]. In Chinese men, an intervention of Tai Chi was effective at reducing BP and TG [77]. Less is known about how similar interventions may be effective in African-American, Hispanic and Aboriginal/Indigenous populations. However, higher levels of physical activity and fitness have been reported to be associated with a lower prevalence of MetS in African-Americans [78], Hispanics [79] and Aboriginals [80].

To complement physical activity interventions, consideration should be given to interventions to limit sedentary behaviour such as sitting. Prospective studies have reported extended sedentary time to be positively associated with increased risk for type 2 diabetes, cardiovascular disease and premature mortality [81]. These associations occur even independently of physical activity levels. Cross-sectional studies in Americans [82], Brazilians [83] and Koreans [84] have reported positive associations between sedentary time and MetS. Randomised interventions aimed at reducing sedentary time have proven successful in increasing physical activity [85,86].

## 4. Prevention

Given the overall numbers of people with, and at risk for, MetS in most countries, individual-based prevention approaches are unlikely to be efficient and feasible. Instead, prevention strategies should be targeted to populations at the community level [87] and must be culturally tailored, as what works in one race/ethnic group may not necessarily work in another. These can vary from upstream, high-level policy initiatives to downstream, on the ground programs targeted at high-risk groups.

Policies such as the introduction of a sugar tax show promise. A high consumption of sugar, and sugar-sweetened beverages in particular, has been associated with a higher prevalence of MetS in a number of countries [88,89]. Countries and regions that have implemented a sugar tax have reported reductions in the consumption of sugar-sweetened beverages [90,91,92]. However, at present, there is no evidence to indicate this translates into a reduction in the incidence of MetS or its components, most likely because these policies are relatively new and may need more time for a downstream effect to be realised.

Another area influenced by policy at the local level is the built environment, which comprises the human-made infrastructure in which we live. This consists of such things as the street network, the placement of stores, community centres and residential areas, as well as the presence of sidewalks. Aspects of the built environment are associated with both physical activity and diet [93] and may be an upstream determinant for MetS.

People living in areas that are considered walkable (such as those with high street connectivity, mixed land use, and sidewalks) have higher physical activity levels and are at lower risk for T2D compared with those living in nonwalkable areas [94]. Similarly, living in an area with a high proportion of fast food restaurants and limited opportunities to buy healthy foods is associated with a greater prevalence of obesity [95]. These findings are consistent with a systematic review, which found cross-sectional associations of the built environment with obesity, hypertension and MetS, such that areas considered more walkable had a lower prevalence of these conditions [96]. Being cross-sectional, these studies cannot provide insight into causal relations or address the possible numerous confounders such as socioeconomic status (SES) and that some people are able to choose their neighbourhood based on their preferred lifestyle, while others may be limited in opportunities of residential movement as a result of their SES and race/ethnic minority status due to historical segregation. A limited number of longitudinal studies have reported that changes in the built environment associated with more walkability have corresponded with increased walking [97,98].

While some studies have indicated that the local food environment is associated with diets of nearby residents [99,100], not all studies have [101]. Similarly, the association of the food environment with risk factors is less clear. Some studies reported a positive association between fast food restaurants and obesity [95,102], a negative association between supermarkets and obesity [103], while others observed no association between food stores and obesity [104]. However, it appears these associations may depend on socioeconomic strata, which in heterogeneous populations often aligns along race/ethnicity, as the positive relationship between fast food restaurants and obesity was strongest in those with the lowest income [100,105]. Whether interventions to change the local food environment affect risk for the MetS is not known. Introduction of a supermarket in an area previously absent of any had a modest effect on diet quality, such as reduced sugar consumption in nearby residents compared with a control neighbourhood [106]. However, dietary assessment was conducted less than a year after the supermarket opened, and it may take a longer time, and more supermarkets, to change food purchasing behaviours.

Of importance is that many people in high- and middle-income countries who are at high risk for MetS are also among those with the lowest SES [107,108]. In addition, in countries with a diverse population, racial/ethnic minorities tend to be the most marginalised in that society. In lower SES communities, there are fewer opportunities for physical activity (such as green areas and community centres), less access to grocery stores and supermarkets (which sell healthy foods) and a higher proportion of fast food restaurants [109,110]. Targeted built environment interventions in these high-risk communities may be worthwhile to improve lifestyle behaviours known to protect against MetS.

While built environment initiatives affect the whole population, targeting interventions in high-risk communities by bringing prevention strategies to places where people gather have demonstrated substantial promise. These types of programs are needed, as access to health services for prevention and treatment (whether physical or cultural) is often worse for those of lower SES and/or of a minority racial/ethnic status [111]. In African-American communities in the United States, this has taken on the form of BP interventions at local barbershops. Both encouragement of lifestyle modification by their barber and integration with onsite pharmacists resulted in significant BP reductions, with the latter intervention being significantly better than barber encouragement alone [112]. Similar intervention studies are ongoing in faith-based communities and places of worship [113].

Success has also been reported using workplace interventions, which have resulted in improvements in activity and nutrition compared with control [114]. In Delhi, a multifactorial six-month worksite intervention focusing on education resulted in improvements in HDL-C, TG and WC compared with control groups [115]. Others have looked at translating successful interventions in European-derived populations to different racial/ethnic groups and delivering them in local communities. These studies have demonstrated significant reduction in MetS risk factors in African-Americans, [116] Hispanics [117,118] and South Asians living in India, Pakistan and the United Kingdom [119,120,121]. Community initiatives have also worked in increasing physical activity through walking programs in Hispanic neighbourhoods [122] and improving nutrition through local dietary counselling in African-American neighbourhoods [123]. Other community-based interventions have reported improvements in HDL-C, BP and WC compared with control in Taiwan [124]. A cluster-randomised study of communes in Vietnam found a six-month physical activity and nutrition intervention also improved cardiometabolic risk factors and a slightly better reduction in prevalence of MetS compared with an educational intervention [125]. In Iran, community-based educational programs have been successful in reducing the incidence of MetS compared with nonintervention controls [126,127].

Another area of promise for individual interventions but on a population scale is the use of consumer technology devices such as tablets, smartphones and wearables. A number of randomised studies have reported on experimental interventions that have improved lifestyle behaviours and/or cardiometabolic risk factors related to MetS, whether through wearable technology, smartphone apps or simple text messaging [29,128,129]. With the increasing ubiquity of global ownership of these devices, the opportunity to leverage these technologies to intervene on a population level has grown. Recent studies have demonstrated the possible effectiveness of large-scale interventions [130] and the feasibility of reach in pragmatic trials [131].

## 5. Other Considerations

While both physical activity and dietary interventions are likely to be efficacious treatments for MetS, the intervention that is effective in one racial/ethnic group may not be effective in a different racial/ethnic group. This can be due to not only different cultural contexts of physical activity and diet but also due to structural barriers and policies, which cater to the majority population and may pose barriers to maintaining health and disease prevention for minority populations. Various cultures also view physical activity in different lights. For example, South Asians have lower physical activity levels compared with other populations [132,133], which may be rooted in cultural context [134]. In addition, adherence to and enjoyment of exercise may be based on the physical activity type, which also may have cultural relevance, such as Bhangra dance in South Asians [73] or Tai Chi in East Asians [135].

Similarly, food is strongly rooted in culture, and availability and cost may differ from place to place. Therefore, interventions need to consider what foods target groups have access to, the cultural meaning of food and the financial opportunity of the individuals. If interventions are not designed taking cultural preferences into account, they are unlikely to be engaged by the population and be successful [136]. In addition to cultural differences, in many high-income countries, many racial/ethnic groups comprise a minority population and are commonly marginalised in society, creating further barriers for treatment. To be effective, interventions must also address real and perceived structural barriers and policies present within each racial/ethnic group. For example, as a result of residential schools in Canada, there is distrust between people of Aboriginal background and government-funded health care [137]. Therefore, trust in the health care system and health care professionals, who may not be from the same community, is needed before effective prevention and intervention can begin [138].

## 6. Future Directions and Conclusions

Despite more than 80% of the world’s population being of non-European descent, the overwhelming majority of research on MetS, from prevalence to treatment, is in predominantly European-derived populations. This is a critical gap in knowledge given that cardiometabolic risk may differ along racial/ethnic lines. Indeed, the recognised different WC thresholds reflect the nuances of race/ethnicity when assessing MetS. Current evidence in prevention and treatment of MetS suggests lifestyle interventions proved in European-derived populations can be effective at treating and reversing MetS; however, they need to be translated into the local cultural context to ensure success. For widespread prevention of MetS, interventions targeted at the population level are likely to be most successful. In order to grow our knowledge of MetS in different populations around the world, we need to conduct more rigorous cohort and randomised trials in populations beyond those of European descent. In addition, studies in countries with significant diversity should include unrepresented racial/ethnic groups as well as analyses stratified by race/ethnicity.

## Figures and Tables

**Table 1:** Criteria of the metabolic syndrome defined as three of more of the five measures.

Measure	Threshold
Elevated triglycerides	≥1.70 mmol/L *
Reduced HDL-C	≤1.00 mmol/L (males) * ≤1.30 mmol/L (females) *
Elevated blood pressure	Systolic ≥ 130 mmHg and/or Diastolic ≥ 85 mmHg *
Elevated fasting glucose	≥5.6 mmol/L *
Elevated waist circumference	See population-specific thresholds in Table 2

* Or appropriate drug treatment. HDL-C = high-density lipoprotein cholesterol.

**Table 2:** Population-specific waist circumference thresholds [2].

Population	Men	Women
Central/South American, Chinese, Japanese, South Asian	≥90 cm	≥80 cm
Mediterranean, Middle East, Sub-Saharan African	≥94 cm	≥80 cm
Europid (includes Canada, Europe and United States) *	≥102 cm (≥94 cm)	≥88 cm (≥88 cm)

* While thresholds of ≥94 cm for men and ≥80 cm for women are more common in research, the thresholds of ≥102 for men and ≥88 for women are used more commonly in clinical practice.
